# DiDang Tang Inhibits Endoplasmic Reticulum Stress-Mediated Apoptosis Induced by Oxygen Glucose Deprivation and Intracerebral Hemorrhage Through Blockade of the GRP78-IRE1/PERK Pathways

**DOI:** 10.3389/fphar.2018.01423

**Published:** 2018-12-04

**Authors:** Qingxia Huang, Tianye Lan, Jing Lu, He Zhang, Dongmei Zhang, Tingting Lou, Peng Xu, Jixiang Ren, Daqing Zhao, Liwei Sun, Xiangyan Li, Jian Wang

**Affiliations:** ^1^Research Center of Traditional Chinese Medicine, Changchun University of Chinese Medicine, Changchun, China; ^2^Jilin Provincial Key Laboratory of BioMacromolecules of Chinese Medicine, Changchun University of Chinese Medicine, Changchun, China; ^3^Department of Encephalopathy, Changchun University of Chinese Medicine, Changchun, China; ^4^Scientific Research Office, Changchun University of Chinese Medicine, Changchun, China; ^5^Jilin Ginseng Academy, Changchun University of Chinese Medicine, Changchun, China

**Keywords:** oxygen and glucose deprivation, endoplasmic reticulum stress (ER stress), DiDang Tang, mitochondrial dysfunction, apoptosis, GRP78-IRE1/PERK pathways

## Abstract

DiDang Tang (DDT), a Chinese traditional medicine formula, contains 4 Chinese traditional medicine substances, has been widely used to treat intracerebral hemorrhage (ICH) patients. However, the molecular mechanisms of DDT for protecting neurons from oxygen and glucose deprivation (OGD)-induced endoplasmic reticulum (ER) stress and apoptosis after ICH still remains elusive. In this study, high-performance liquid chromatography fingerprint analysis was performed to learn the features of the chemical compositions of DDT. OGD-induced ER stress, Ca^2+^ overload, and mitochondrial apoptosis were investigated in nerve growth factor -induced PC12, primary neuronal cells, and ICH rats to evaluate the protective effect of DDT. We found that DDT treatment protected neurons against OGD-induced damage and apoptosis by increasing cell viability and reducing the release of lactate dehydrogenase. DDT decreased OGD-induced Ca^2+^ overload and ER stress through the blockade of the glucose-regulated protein 78 (GRP78)- inositol-requiring protein 1α (IRE1)/ protein kinase RNA-like ER kinase (PERK) pathways and also inhibited apoptosis by decreasing mitochondrial damage. Moreover, we observed similar findings when we studied DDT for inhibition of ER stress in a rat model of ICH. In addition, our experiments further confirmed the neuroprotective potential of DDT against tunicamycin (TM)-induced neural damage. Our *in vitro* and *in vivo* results indicated that the neuroprotective effect of DDT against ER stress damage and apoptosis occurred mainly by blocking the GPR78-IRE1/PERK pathways. Taken together, it provides reliable experimental evidence and explains the molecular mechanism of DDT for the treatment of patients with ICH.

## Introduction

Oxygen and glucose deprivation (OGD) of the neurons surrounding a hematoma has been considered a major factor in neuronal cell death in acute and chronic intracerebral hemorrhage (ICH) (Chien et al., 2015; Selim and Sheth, 2015; Korompoki et al., 2017). During an ICH, blood rushes into the surrounding brain tissue, at high pressure, resulting in brain damage from reduced regional cerebral blood flow in the perihematoma, and directly causing neuronal ischemia and hypoxia (Karaszewski et al., 2015; Rickards, 2015; Vavilis et al., 2015). Increasing evidences has shown that under OGD after ICH, protein misfolding and accumulation in the endoplasmic reticulum (ER) lumen initiate ER stress (Cominacini et al., 2015) and lead to mitochondrial apoptosis (Yuan et al., 2017). Therefore, the suppression of ER stress-mediated apoptosis could be a potential and effective strategy for the treatment of ICH (Zhang et al., 2015).

External environment changes, such as energy deprivation, cause the accumulation of misfolded proteins in the ER lumen (Zhong et al., 2013). Glucose-regulated protein 78 (GRP78), an ER chaperone, detects the accumulation of unfolded proteins and activates three main type-I transmembrane proteins, namely, inositol-requiring protein 1α (IRE1), activating transcription factor-6 (ATF6), and protein kinase RNA-like ER kinase (PERK), to initiate the unfolded protein response (UPR) (Cominacini et al., 2015). Once the UPR fails to control the level of unfolded and misfolded proteins in the ER, ER-initiated apoptotic signaling is induced (Roussel et al., 2013). The ER is known as a major intracellular Ca^2+^ storage compartment and plays a critical role in the maintenance of cellular Ca^2+^ homeostasis (Tusi et al., 2011). In central nervous system diseases, sustained ER stress results in a release of Ca^2+^ into the cytosol (Belal et al., 2012). Free Ca^2+^ in the cytosol can be either transported to the mitochondria or used to activates cellular apoptosis pathways directly (Duan et al., 2017), This is critically regulated by the Bcl-2 family (Gupta et al., 2010). Therefore, we speculate that OGD after ICH leads to neuronal apoptosis by triggering ER stress and Ca^2+^ overload.

DiDang Tang (DDT) originates from *Treatise on Cold Pathogenic Diseases*, a Chinese traditional medicine formulary. It contains 2 Chinese traditional medicine plants, including *rhubarb* and *peach seed*, and 2 Chinese traditional medicine animals, *leech*, and *gadflies*, and it has been widely used to treat ICH patients and animal models (Li et al., 2015; Xu et al., 2015, 2017). Our previous study showed that DDT significantly reduced brain water content and intracerebral hematoma volume in rats with ICH by up-regulating the expression of brain-derived neurotrophic factor, tyrosine kinase B and vascular endothelial growth factor (VEGF) (Ren et al., 2013). In addition, previous reports showed that *leech* alcohol extract and *rhubarb* water extract alleviated the inflammation of peripheral tissue and cerebral edema in rats with ICH (Li et al., 2014; Wang et al., 2016). *Peach seed* water extract up-regulated VEGF and VEGF receptor 2 after ICH in a mouse model (Cui et al., 2015) and inhibited glucose deprivation injury in PC12 cells (Qi et al., 2014). However, the molecular mechanisms of DDT for protecting neurons from OGD-induced ER stress and apoptosis after ICH still remains elusive. In this study, we investigate the protective effect of DDT on OGD-induced ER stress, Ca^2+^ overload, and mitochondrial apoptosis and reveal the molecular mechanism of DDT on blocking GRP78- IRE1/PERK pathways in PC12 and primary neuronal cell models of ER stress and apoptosis induced by OGD or tunicamycin (TM), an ER stress inducer, and in a rat model of ICH, which we call the ICH rat.

## Materials and Methods

### Materials and Reagents

4-PBA (P21005) and Hoechst 33342 (B2261) were purchased from Sigma-Aldrich (St. Louis, MO, United States), dissolved in dimethyl sulfoxide (DMSO) and stored at -20°C before use. TM (TM, ab120296) and antibodies against GRP78 (ab21685), IRE1 (ab37073), and phospho-IRE1 (Ser724, ab48187) were obtained from Abcam (Cambridge, MA, United States). Antibodies against microtubule-associated protein 2 (MAP2, #4542), eukaryotic translation initiation factor 2α (eIf2α, #5324), phospho-eIf2α (Ser51, #3398), PERK (#3192), phospho-PERK (Thr980, #12185), Bax (#2772), Bcl-2 (#2870), Cytochrome C (Cyto C, #4280), Bad (#9292), phospho-Bad (Ser112, #9291), PARP (#9532), and β-Actin (#3700) were purchased from Cell Signaling Technology (Beverly, MA, United States). Antibody against ATF6 (IMG273) was purchased from Novus Biologicals (Littleton, CO, United States).

### Preparation of DDT and HPLC Fingerprint Analysis

DiDang Tang was provided by the Department of Pharmacy, the Affiliated Hospital to Changchun University of Traditional Chinese Medicine (Jilin, China) and included the four components (Supplementary Table 1) at a weight ratio of 5:3:10:3. The voucher specimens were deposited at the Research Center of Traditional Chinese Medicine, the Affiliated Hospital to Changchun University of Chinese Medicine. The 21.0 g powders of DDT were decocted with 40 ml water at 100°C for 1 h three times to obtain the aqueous extract according to the standard procedures (National Pharmacopoeia Committee, 2005). All of the solutions after decoction were combined and centrifuged, and the supernatants were dried under vacuum to produce a brownish powder with a yield of 21.5%. The extract was stored at -80°C until use. For biological studies, 0.1 g/ml stock concentration decoction of DDT was filtered (0.2 μm), sterilized, and diluted as previously described (Yan et al., 2015).

**FIGURE 1:**
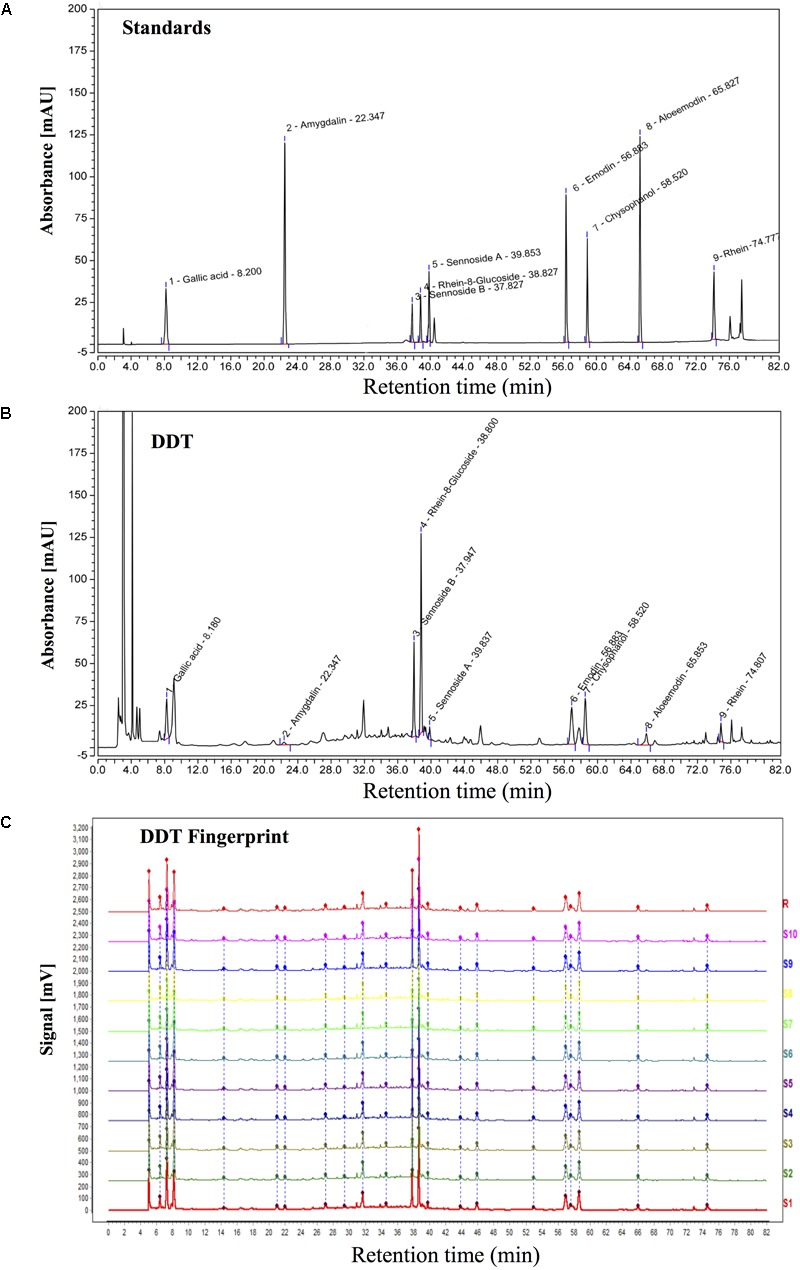
HPLC chromatogram of DiDang Tang (DDT). **(A)** HPLC chromatograms of gallic acid, amygdalin, sennoside B, rhein-8-glucoside, sennoside A, emodin, chrysophanol, aloe-emodin and rhein with UV detection at 254 nm are shown. **(B)** HPLC chromatogram of DDT with UV detection at 254 nm is shown. **(C)** HPLC fingerprint chromatograms of 10 batches of DDT (S1–10) and the control fingerprint chromatogram (R) from the analysis of 10 batches of DDT using National Pharmacopeia Committee Chinese Medicine Fingerprint Similarity Evaluation System (2004A) Software with UV detection at 254 nm are shown.

Ten different batches of DDT were separated by a ZORBAX SB-C18 column (4.6 × 250 mm, 5 μm, Agilent, Santa Clara, CA, United States) and analyzed for specific ingredients and chemical fingerprints using high-performance liquid chromatography (HPLC, Agilent) and a diodearraydetector (DAD) detector (Ultimate3000, DIONEX, Sunnyvale, CA, United States) (Wang et al., 2012; Zhang et al., 2017). The mobile phase condition was recorded as follows: acetonitrile (AS1122-801, TEDIA) in water (A) and 0.1% acetic acid in water (B). The column temperature was 25°C, and the flow rate was 0.9 ml/min, with UV detection at 254 nm. Acquisition and analysis of chromatographic data were performed using Empower software (Agilent). Gallic acid, amygdalin, sennoside B, rhein-8-glucoside, sennoside A, emodin, chrysophanol, aloe-emodin and rhein from China Food and Drug Research Institute were used as surrogate markers (Figure 1A). Eighteen major peaks of DDT extract were identified on the HPLC fingerprints and nine peaks were identified by comparing the retention times with the DDT (Figure 1B). As shown in Figure 1C, the similarities were found to be within the range of 97% to 99% overall for all of the 10 batches analyzed, suggesting that the overall quality of our DDT has good reproducibility.

### Cell Culture and OGD Modeling

PC12 cells were obtained from American Type Culture Collection (ATCC, Manassas, VA, United States) and maintained in RPMI 1640 medium (Gibco, New York, NY, United States) supplemented with 7.5% fetal bovine serum (FBS, CLARK Bioscience, Claymont, DE, United States), 2.5% horse serum (Gibco), 100 units/ml penicillin (Biosharp, Hefei, China), and 100 μg/ml streptomycin (Biosharp) at 37°C in a humidified atmosphere with 5% CO_2_. PC12 cells were incubated with 50 ng/ml nerve growth factor (NGF, 13257019, Gibco) for 24 h to induce neurite formation as a model of neuronal cells (Zhong et al., 2012). Primary cortical neurons were isolated from day 16–18 rat embryos (Experimental Animals Center, Jilin University, Jilin, China) as previously described (Ni et al., 2015 Luo et al., 2017). Briefly, cortices were dissected, mechanically minced and then dissociated by 0.25% trypsinization at 37°C for 20 min. After centrifugation at 1000 rpm for 5 min, each single cell suspension was resuspended in DMEM medium (Gibco) with 10% FBS, 100 units/ml penicillin and 100 μg/ml streptomycin and incubated at 37°C in a water-saturated atmosphere of 5% CO_2_ for 24 h. After attachment, primary neurons were cultured in neurobasal medium (Gibco) with 2% B-27 (Gibco) and 0.5 mM Glutamax (Gibco) for 8 days before further experiments and were identified by the main neuronal marker, MAP2. To establish OGD-induced cell injury models, PC12 cells or primary neurons were kept in serum- and glucose-free medium and incubated in an oxygen free (95% N_2_, 5% CO_2_) BioSpa automated incubator (BioTek, Winooski, VT, United States) for 0.5–2.5 or 2–10 h, respectively (Yan et al., 2015; He et al., 2016).

### Animal Studies

Adult male Sprague–Dawley rats (250–300 g) were obtained from the Experimental Animal Center of Jilin University in Changchun, China and were housed in an animal care facility under a 12 h light/dark cycle. Animal care and surgical procedures were performed in accordance with guidelines approved by the Experimental Animals Committee of Changchun University of Chinese Medicine, Changchun, China for the ethical use of animals. In this study, rats were numbered and randomly divided into sham procedure group (Sham), an ICH-vehicle group (ICH), and an ICH-DDT group (ICH + DDT, 1.3 g/kg, twice a day for 3 days at 2 h after ICH). The ICH models were established as previously described (Caliaperumal and Colbourne, 2013; Kim et al., 2015). Briefly, 10 rats for the sham procedure and 20 rats for ICH were anesthetized with isoflurane and placed in a stereotaxic frame. A hole was drilled (3 mm to the right and 0.4 mm posterior of the bregma) and 50 μL of 0.9% sterile saline or autologous blood was infused over 5 min through a 26-gauge needle (Hamilton syringe, Reno, NV, United States) at a depth 6.5 mm below the skull. The hole was sealed with bone wax and the wound was sutured. Body temperature was maintained at 37°C throughout the procedure, and the rats were given free access to food and water after they woke up. Neurological severity of rats in each group was evaluated using the Longa neurological severity score as follows: grade 0, no neurologic deficit; grade 1, failure to extend left forepaw fully; grade 2, circling to the left; grade 3, falling to the left; grade 4, unable to walk spontaneously; thus, the higher score, the more severe the injury. The scoring was assessed by two non-experimental personnel following the double-blind principle (Feng et al., 2018; Li et al., 2018).

### Immunohistochemical Staining and TUNEL Assay

Immunohistochemical (IHC) staining and TUNEL assay (C1090, Beyotime Biotechnology) were performed in brain tissues fixed with 4% paraformaldehyde, paraffin-embedded, and sectioned as described previously (Ni et al., 2015). The brain tissues from different groups were analyzed for GRP78 (1:100) expression. The percentages of TUNEL-positive cells were calculated in 5 randomly selected microscopic fields at a 400 × magnification.

### Cell Viability Assay

After treatment with DDT or 4-PBA for 48 h prior to OGD or TM incubation, MTT at 0.5 mg/ml in PBS was added to each well to determine cell viability. The formazan crystals were dissolved with 150 μl of DMSO, and the absorbance was detected at 570 nm on a microplate reader (Infinite M200Pro, Tecan, Zürich, Switzerland) as described previously (Chen et al., 2017).

### Annexin V/PI Staining

Cell apoptosis was analyzed using Annexin V-FITC and Propidium Iodide (PI, Beyotime Biotechnology, Shanghai, China) staining according to the manufacturer’s instructions (Ma et al., 2014). After the treatment with DDT or 4-PBA for 48 h prior to OGD or TM incubation, the cells were collected by centrifugation at 1500 rpm for 5 min. After removal of the supernatant, cells were incubated in 50 μl of binding buffer containing 5 μl Annexin V-FITC in the dark for 15 min, and then 10 μl PI was added in the dark at room temperature for 5 min. After adding 400 μl of binding buffer, the percentages of apoptotic cells from 10,000 cells per sample were analyzed using a flow cytometer (FACS Calibur^TM^, BD Biosciences, CA, United States).

### Mitochondrial Membrane Potential Measurement

Mitochondrial membrane potential (MMP) was measured by a JC-1 florescent probe (Beyotime Biotechnology), according to the manufacturer’s instructions (Zhong et al., 2018). The cells were cultured in 6-well plates (1 × 10^5^/well) and pretreated with various concentrations of DDT for 48 h. After OGD incubation, the cells were stained with JC-1 in the dark at 37°C for 30 min, and observed under a fluorescence microscope or analyzed by a flow cytometer (BD Biosciences).

### Western Blot Analysis

Cells or brain tissues were lysed in RIPA buffer (Beyotime Biotechnology) for 30 min on ice, and protein concentration was quantified with a BCA protein assay kit (Beyotime Biotechnology). After boiling at 100°C for 5 min, proteins (50 μg) were subjected to 10% or 12% SDS–PAGE gels and transferred onto PVDF membranes as described previously (Eckle et al., 2012). After blocking with 5% non-fat milk, membranes were probed with primary antibodies overnight at 4°C. β-Actin (1:1000) was used as a loading control. After incubation with appropriate secondary antibodies for 1 h at room temperature, protein bands were visualized and analyzed using a chemiluminescent imaging system (FluorChem, ProteinSimple, San Jose, CA, United States).

### Immunofluorescence Staining

PC12 cells and primary neurons were placed in 12-well chamber slides (NEST Biotechnology, Shanghai, China). After treatment with DDT and an OGD incubation period, the cells were fixed in 4% formaldehyde and incubated with primary antibodies (1:200) overnight at 4°C. After the incubation with Cy3-labeled goat anti-mouse or FITC-labeled goat anti-rabbit antibodies (1:1000, BOSTER, Wuhan, China) for 1 h, the cells were mounted with medium containing DAPI for 10 min to stain nuclei as described previously (Tsushima et al., 2017). A Nikon C2 confocal microscope with ZEN software (Nikon, Tokyo, Japan) was used to analyze the levels of GRP78 and MAP2. A minimum of three cover slips were used for each experimental group.

### Intracellular Calcium Level Measurement

The cells treated with various concentrations of DDT prior to OGD were equilibrated in 5 mM Fura-2/AM (Beyotime Biotechnology) in PBS for 1 h at 37°C and washed with PBS three times as described previously (Seidlmayer et al., 2015). Analysis of intracellular calcium level was performed by flow cytometry (FCM) on a FACS-Calibur (BD Biosciences, CA, United States).

### Statistical Analysis

Results are presented as the mean ± SD from three independent experiments. Statistical significance was analyzed with GraphPad Prism 6 (GraphPad Software, San Diego, CA, United States). Statistical analysis was conducted by using unpaired student *t*-tests for the comparison of two groups or one-way ANOVA for the comparison of several groups. For all of the statistical analyses, a significant difference was accepted when *P* < 0.05.

## Results

### OGD Induces ER Stress and Apoptosis in NGF-Induced PC12 Cells and Primary Culture Neurons

**FIGURE 2:**
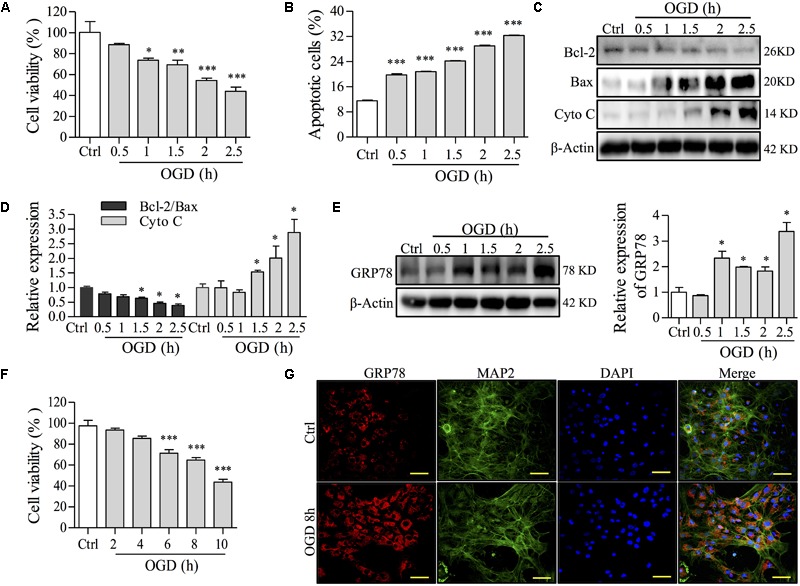
The effects of OGD time on cell damage and ER stress in NGF-induced PC12 cells and primary culture neurons. **(A)** The effect of OGD-induced damage on cell viability was determined by an MTT assay in PC12 cells. **(B)** Apoptosis induced by OGD was determined with Annexin-V/PI staining followed by FCM analysis in PC12 cells. Percentages of apoptotic cells are shown. **(C)** After OGD incubation for the indicated time, the levels of Bcl-2, Bax, Cyto C and β-Actin in PC12 cells were analyzed by Western blot. β-Actin was a loading control. **(D)** Relative expressions of the Bcl-2/Bax ratio and Cyto C form **(C)** were analyzed and are shown. **(E)** GRP78, a central regulator for ER stress, after OGD incubation for different times, was observed by Western blot in PC12 cells. Relative GRP78 expression is shown on the right. **(F)** Primary neuron viability was assayed by an MTT assay after OGD incubation for 2, 4, 6, 8, or 10 h. **(G)** GRP78 expression by immunofluorescence staining after OGD for 8 h in primary neurons. MAP2 is a marker for neurons. Nuclei were stained with DAPI. Scale bar = 50 μm. Ctrl: control group; PC12 cells or primary culture neurons were incubated in normal medium under normoxia (37°C, 5% CO_2_) in each experiment. ^∗^*P* < 0.05, ^∗∗^*P* < 0.01, and ^∗∗∗^*P* < 0.001 vs. Ctrl group (*n* = 3).

To establish OGD cell models, PC12 cells and primary culture neurons were incubated with no glucose/FBS medium under oxygen-free conditions (95% N_2_, 5% CO_2_) for different times. After OGD induction, cell viability, apoptosis and ER stress were investigated by MTT, FCM, Western blot, and immunofluorescence staining assays to determine an optional condition. In the PC12 cell model of OGD injury, OGD decreased cell viability and increased apoptosis in a time-dependent manner unlike cells cultured in regular medium under noxomia (37°C, 5% CO_2_) as a control group (Figures 2A,B). To further confirm the time of OGD incubation in PC12 cell apoptosis, we examined the expression of apoptosis-related proteins, such as Bcl-2, Bax and Cytochrome C (Cyto C), a mitochondrial dysfunction marker. As shown in Figures 2C,D, OGD obviously decreased the ratio of Bcl-2/Bax and increased the expression of Cyto C compared with the control group in a time-dependent manner in PC12 cells. To observe whether the apoptosis induced by OGD was mediated by ER stress, we analyzed the expression of GRP78, which dissociates from transmembrane proteins to trigger the UPR when ER stress occurs (Wang et al., 2017). As shown in Figure 2E, the level of GRP78 during the period of OGD from 1 to 2.5 h, especially for the OGD incubation for 1.5 h was markedly increased compared with the control group of PC12 cells. In addition, OGD caused a decrease of primary neurons viability (Figure 2F) and also up-regulated the expression of GRP78 in the primary culture neuron with positive staining of microtubule-associated protein 2 (MAP2), a marker of neuronal cells (Figure 2G). These results indicated that OGD caused ER stress and led to cell apoptosis in both models. Based on the results above, we confirmed that the incubation with no glucose/FBS medium for 1.5 h in PC12 cells or for 8 h in primary neurons (model group) was selected as an optimal condition for the following experiments.

### DDT Protects Neuronal Cells From OGD-Induced Cell Damage and Apoptosis in NGF-Induced PC12 Cells and Primary Neurons

**FIGURE 3:**
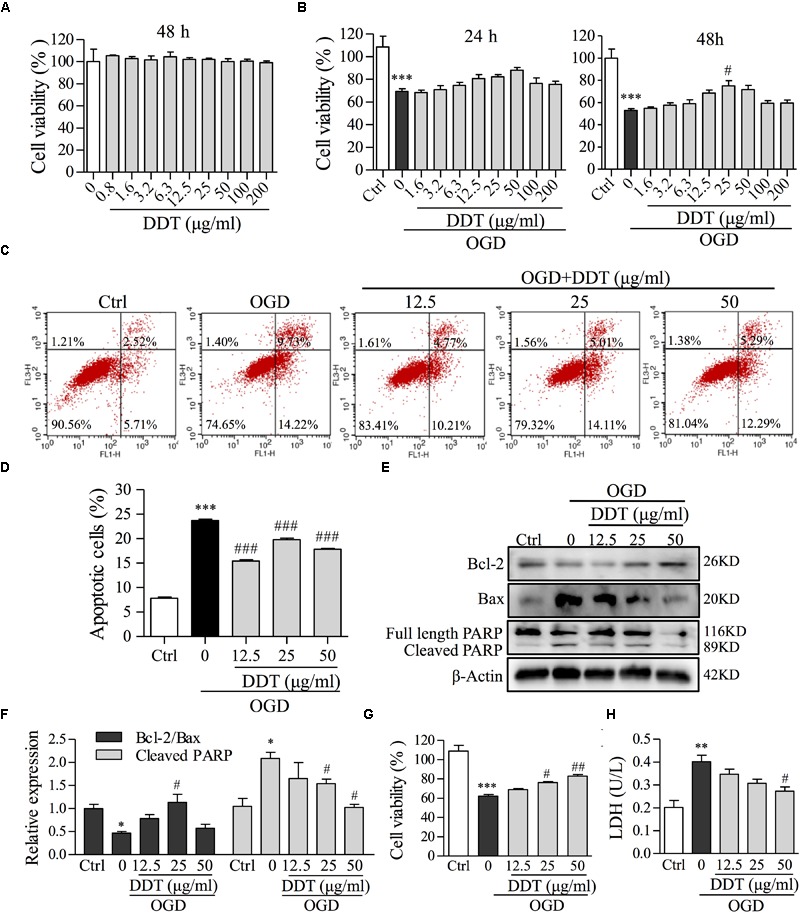
The effects of DDT pretreatment on OGD-induced cell viability and apoptosis in NGF-induced PC12 cells and primary culture neurons. **(A)** PC12 cells were treated with the indicated concentrations of DDT for 48 h. Cell viability was assessed using an MTT assay after treatment. **(B)** PC12 cells were treated with DDT for 24 h or 48 h, followed by OGD for 1.5 h and cell viability was assessed by an MTT assay. **(C)** PC12 cells pretreated with DDT at the concentrations of 12.5, 25, or 50 μg/ml for 48 h, followed by OGD for 1.5 h, were stained with Annexin V and PI, and then analyzed by flow cytometry (FCM). **(D)** Data are expressed as the average percentages of apoptotic cells from **(C)**. **(E)** PC12 cells treated with DDT and incubated with OGD exposure were collected and lysed. The levels of Bcl-2, Bax, and PARP were analyzed by Western blot. β-Actin was the loading control. **(F)** The values represent the ratio of Bcl-2/Bax and the cleavage of PARP quantified by Image J from **(E)**. **(G)** The viability of primary neurons treated with DDT for 48 h, followed by OGD for 8 h, was assessed by an MTT assay. **(H)** The effect of DDT for 48 h on the release of LDH in culture medium from primary neurons after 8 h incubation in OGD was determined using an LDH assay kit. ^∗^*P* < 0.05, ^∗∗^*P* < 0.01, and ^∗∗∗^*P* < 0.001 vs. Ctrl group; ^#^*P* < 0.05, ^##^*P* < 0.01, and ^###^*P* < 0.001 vs. OGD group (*n* = 3).

To evaluate the neuroprotective effect of DDT, PC12 cells and primary cultures of neurons were treated with different doses of DDT, and exposed to OGD incubation. Cell viability was first measured to observe cytotoxic effects of DDT in PC12 cells. As shown in Figure 3A, DDT pretreatment for 48 h had no cytotoxic effects in PC12 cells below 200 μg/ml. In the OGD-induced PC12 cell model, DDT pretreatment at the concentration of 12.5–50 μg/ml for 24 or 48 h increased cell viability induced by OGD in a concentration- and time-dependent manner (Figure 3B). FCM analysis showed that DDT pretreatment for 48 h obviously inhibited OGD-induced cell apoptosis (Figures 3C,D). The anti-apoptosis mechanism of DDT was further detected by Western blot analysis. Pretreatment with DDT increased the ratio of Bcl-2/Bax and decreased the cleavage of PARP, compared to the OGD model group (Figures 3E,F). Additionally, DDT pretreatment also increased cell viability and obviously inhibited the release of LDH induced by OGD incubation in a dose-dependent manner in the primary culture neurons as shown in Figures 3G,H. The results indicated that preconditioning with DDT could protect neurons against OGD-induced damage and apoptosis.

### DDT Decreases Ca^2+^ Overload and ER Stress Induced by OGD Through Blocking IRE1 and PERK Signaling in NGF-Induced PC12 Cells and Primary Neurons

**FIGURE 4:**
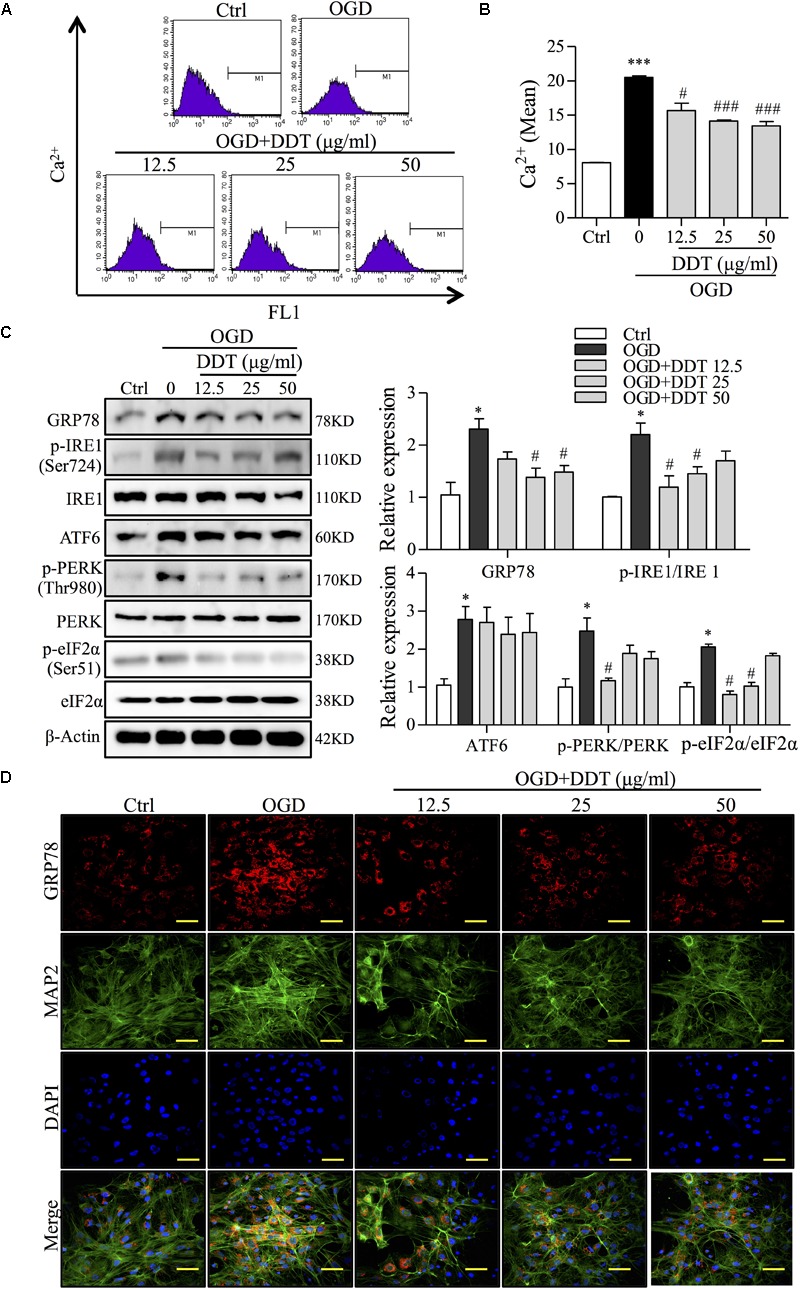
DiDang Tang inhibits Ca^2+^ overload and ER stress in NGF-induced PC12 cells and primary culture neurons subjected to OGD. **(A)** Intracellular Ca^2+^ levels were evaluated by FCM using the fluorescent Ca^2+^ indicator, Flou4-AM. **(B)** Bar graph represents the fluorescence intensity of Flou4-AM. **(C)** PC12 cells after the treatment of DDT for 48 h and an OGD incubation for 1.5 h were prepared as described in Materials and Methods and resolved by SDS–PAGE followed by Western blot using antibodies against GRP78, p-IRE1 (Ser724), IRE1, ATF6, p-PERK (Thr980), PREK, p-eIf2α (Ser51), eIf2α, and β-Actin. Relative expression was quantified with Image J and is shown on the right. **(D)** The expressions of GRP78 and MAP2 in primary neurons treated with DDT at different concentrations for 48 h, followed by OGD for 8 h were detected by immunofluorescence staining. Nuclei were stained with DAPI. Scale bar = 50 μm. ^∗^*P* < 0.05 and ^∗∗∗^*P* < 0.001 vs. Ctrl group; ^#^*P* < 0.05 and ^###^*P* < 0.001 vs. OGD group (*n* = 3).

The normal ER functions to regulate and control intracellular Ca^2+^, thus governing protein synthesis, and the ER disturbance results in a release of Ca^2+^ into the cytosol and cell apoptosis (Shinde et al., 2016). An analysis of fluorescence intensity of Ca^2+^ by FCM in PC12 cells showed that OGD induced intracellular Ca^2+^ overload, which was partially prevented by DDT treatment (Figures 4A,B). To restore ER homeostasis, GRP78 dissociates from UPR proteins to induce the activation of three transmembrane proteins, IRE1, ATF6, and PERK (Cominacini et al., 2015) to correct folding and eliminate faulty protein. However, the ER stress occurs when there is fails to control the level of unfolded proteins (Hetz and Saxena, 2017). To reveal the mechanism of DDT in ER stress induced by OGD, we performed Western blot analysis for the expressions of GRP78, three transmembrane proteins, and the PERK downstream target, eIF2α (Bravo et al., 2013). As shown in Figure 4C, the levels of GRP78 and the UPR proteins were all increased after OGD incubation compared to the control group, indicating that ER stress was induced by OGD. Importantly, DDT pretreatment greatly decreased the expression of GRP78 and the phosphorylation of IRE1 (Ser724) and PERK (Thr980), and did not significantly alter the expression of ATF6 compared to OGD model group. The phosphorylation of eIF2α at Ser51 induced by the OGD was obviously inhibited by DDT pretreatment. In addition, we found that DDT significantly inhibited GRP78 expression induced by OGD for 8 h in the primary culture neurons (Figure 4D).

### DDT Inhibits OGD-Induced Apoptosis by Decreasing Mitochondrial Damage in NGF-Induced PC12 Cells

**FIGURE 5:**
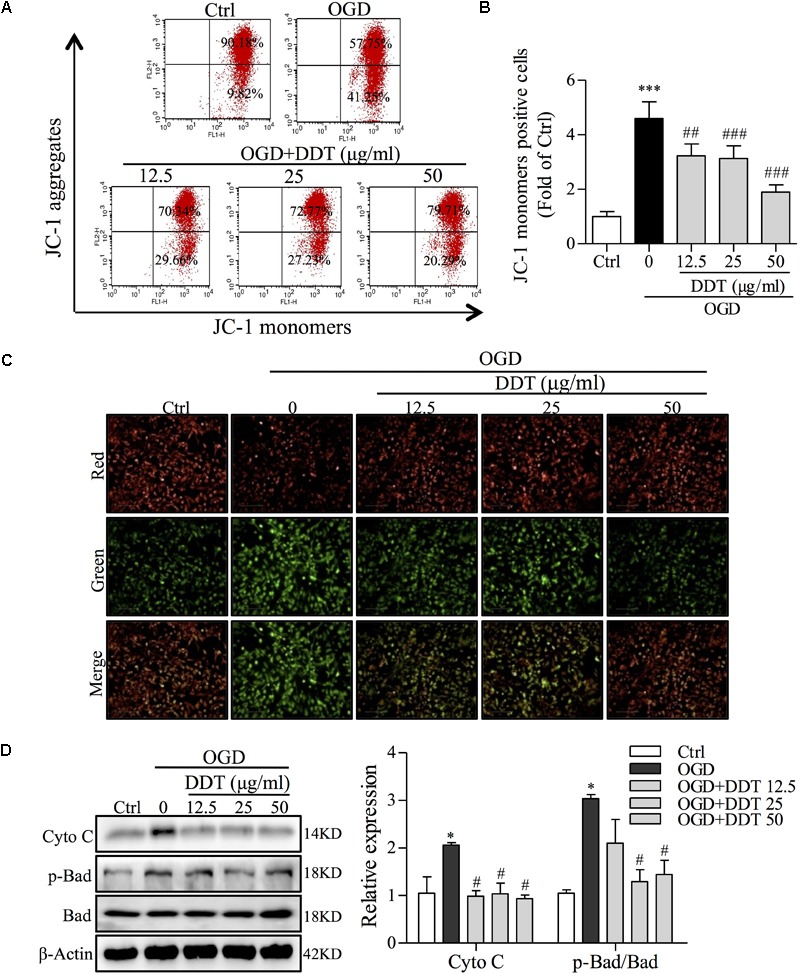
Effects of DDT on OGD-induced intracellular mitochondrial damage in NGF-induced PC12 cells. **(A)** After treatment with DDT prior to OGD for 1.5 h, PC12 cells were incubated with a JC-1 probe to assess mitochondrial membrane potential (MMP) by FCM. **(B)** Bar graph represents the cells positive for JC-1 monomer analyzed from **(A)**. **(C)** MMP of PC12 cells treated with DDT for 48 h and incubated with OGD for 1.5 h was monitored by measuring fluorescence intensity. **(D**) After pretreatment with DDT at different concentrations for 48 h and OGD incubation for 1.5 h, the levels of Cyto C, p-Bad and Bad in PC12 cells were detected by Western blot. ^∗^*P* < 0.05 and ^∗∗∗^*P* < 0.001 vs. Ctrl group; ^#^*P* < 0.05, ^##^*P* < 0.01, and ^###^*P* < 0.001 vs. OGD group (*n* = 3).

Previous studies have shown that ER stress enhances the sequestration of vast amounts of Ca^2+^ in mitochondria to trigger mitochondrial membrane permeabilization and dysfunction (Shinde et al., 2016). To clarify whether the neuroprotective effect of DDT occurs via ER stress-induced mitochondrial dysfunction, we detected MMP and mitochondrial apoptosis pathway-related proteins by FCM and Western blot analyses. As shown in Figures 5A–C, in PC12 cells, OGD induced an increase of JC-1 monomers-positive cells, and DDT at different concentrations significantly decreased the number of these cells induced by OGD. Furthermore, Western blot analysis revealed that OGD increased the levels of Cyto C and p-Bad/Bad in PC12 cells, which were antagonized by DDT pretreatment (Figure 5D). Taken together, these results suggest that DDT decreased mitochondrial dysfunction to recover the damage from OGD-mediated ER stress in neuronal cells.

### DDT Decreases ER Stress Induced by ICH Through Blocking IRE1 and PERK Signaling in Rats

**FIGURE 6:**
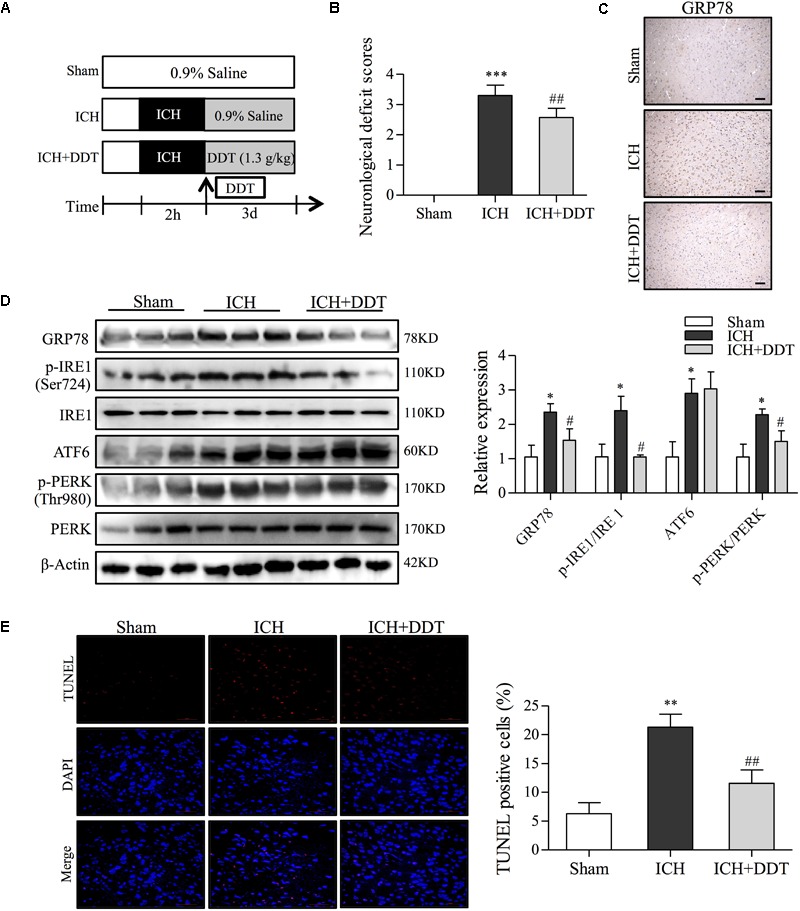
DiDang Tang inhibits ER stress induced by ICH in a rat model. **(A)** Diagram of the animal study for the ICH model or the DDT treatment groups. **(B)** Neurological severity scores of rats in different experimental groups as described in Materials and Methods. **(C)** IHC staining results showed that DDT treatment inhibited the expression of GRP78 in the brain tissues around the bleeding area, compared with the ICH group. **(D)** The ER stress-related proteins in brain tissues from Sham, ICH, and ICH + DDT groups were analyzed by Western blot using antibodies against GRP78, p-IRE1 (Ser724), IRE1, ATF6, p-PERK (Thr980) and PREK. β-Actin was a loading control. Relative expression was quantified with Image J and is shown on the right. **(E)** Apoptotic cells within brain tissues were evaluated by TUNEL assays. Nuclei are counterstained with DAPI (blue). DDT treatment significantly decreased the number of apoptotic cells compared with the ICH group. Quantification of TUNEL-positive cells is shown on the right. ^∗^*P* < 0.05 and ^∗∗∗^*P* < 0.001 vs. Sham group; ^#^*P* < 0.05 and ^##^*P* < 0.01 vs. ICH group (*n* = 5).

To further investigate the protective effect of DDT against ICH-induced ER stress *in vivo*, neurological deficit scores and the effect of DDT on ER stress in ICH rats from vehicle- or DDT-treatment groups were evaluated by a series of experiments. As shown in Figures 6A,B, the Longa neurological deficit score was 0 in the sham group and 3.3 in the ICH group. The rats from the ICH group showed different degrees of failure by circling to the left, falling to the left, and being unable to walk spontaneously. DDT treatment significantly improved the symptoms of the left forepaw circling and falling to the left, which suggested that DDT reduces the injury from ICH. IHC staining of GRP78 showed that DDT significantly inhibited the expression of GRP78 induced by ICH in the brain tissues around the bleeding (Figure 6C). As shown in Figure 6D, Western blot analysis showed that the levels of GRP78, p-IRE1/IRE1, ATF6, and p-PERK/PERK in the brain tissues of ICH rats were obviously higher than in the Sham group. Compared to the ICH group, DDT treatment significantly decreased the expression of GRP78 and the phosphorylation of IRE1 (Ser724) and PERK (Thr980), but did not significantly alter the expression of ATF6 (Figure 6D). In addition, we found that DDT reduced the percentage of apoptotic cells by TUNEL staining compared to the ICH group (Figure 6E). The data above strongly suggest that the neuroprotective effect of DDT *in vivo* was mediated through the inhibition of ER stress-mediated apoptosis through blocking the GRP78-IRE1/PERK pathway, which is consistent with the *in vitro* findings.

### DDT Inhibits TM-Induced ER Stress Damage in NGF-Induced PC12 Cells

**FIGURE 7:**
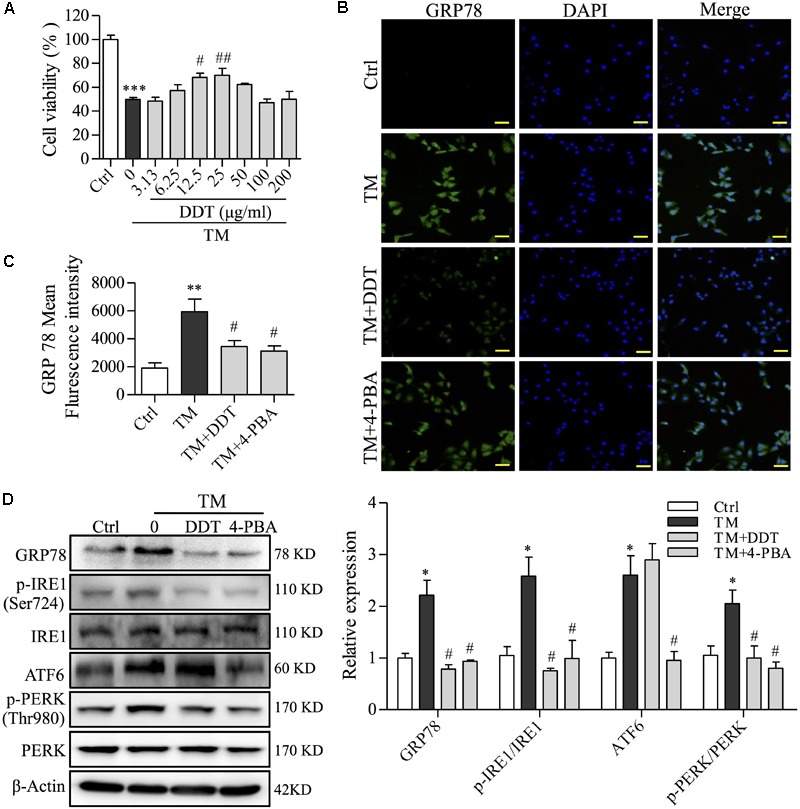
DiDang Tang inhibits TM-induced ER stress damage in NGF-induced PC12 cells. **(A)** The viability of PC12 cells treated with DDT following TM incubation was determined by an MTT assay. **(B)** PC12 cells were treated with DDT or 4-PBA for 48 h, followed by TM incubation, and were stained with GRP78. Nuclei were stained with DAPI. Scale bar = 50 μm. **(C)** The bar graph represents fluorescence intensity of GRP78 expression from **(B)**. **(D)** PC12 cells were treated with DDT or 4-PBA for 48 h prior to TM incubation. The levels of GRP78, p-IRE1, IRE1, ATF6, p-PERK, and PERK were examined by Western blot, and their relative expression was quantified by the means of densitometry analyses on the right. β-Actin was a loading control. An ER stress inhibitor, 4-PBA (250 μM) was used as a positive control. ^∗^*P* < 0.05, ^∗∗^*P* < 0.01, and ^∗∗∗^*P* < 0.001 vs. Ctrl group; ^#^*P* < 0.05 and ^##^*P* < 0.01 vs. TM group (*n* = 3).

To confirm the neuroprotective effect of DDT on the inhibition of ER stress in PC12 cells, we used TM (50 μg/ml) to establish an ER stress cell model (Guha et al., 2017). 4-phenylbutyric acid (4-PBA) was used as a positive control for ER stress inhibition (Guo et al., 2017). TM incubation for 12 h reduced PC12 cell viability to 49.2% compared to the control group. This lost viability was recovered by DDT pretreatment (Figure 7A). To further investigate the molecular mechanisms of DDT underlying TM-induced ER stress, the expressions of GRP78 and three UPR proteins were detected by immunofluorescence staining and Western blot. As shown in Figures 7B,C, the fluorescence intensity of GRP78 in cytoplasm was significantly increased in the TM model group to 3.12-fold compared with the control group, while 4-PBA (250 μM) or DDT (25 μg/ml) pretreatment dramatically reduced the level of GRP78 in PC12 cells. As expected, the levels of GRP78, p-IRE1/IRE1, ATF6 and p-PERK/PERK were increased in PC12 cells after TM incubation, although these levels had been reduced by 4-PBA. DDT pretreatment at a concentration of 25 μg/ml significantly decreased the levels of GRP78, p-IRE1/IRE1 and p-PERK/PERK induced by TM to the control level, but had no effect on ATF6 expression, unlike 4-PBA (Figure 7D), which is consistent with the results presented in Figure 4C. These results indicated that DDT pretreatment obviously reduced the damage of ER stress induced by TM in PC12 cells via inhibition of the GRP78-IRE1/PERK pathways.

### DDT Inhibits TM-Induced Mitochondrial Apoptosis in NGF-Induced PC12 Cells

**FIGURE 8:**
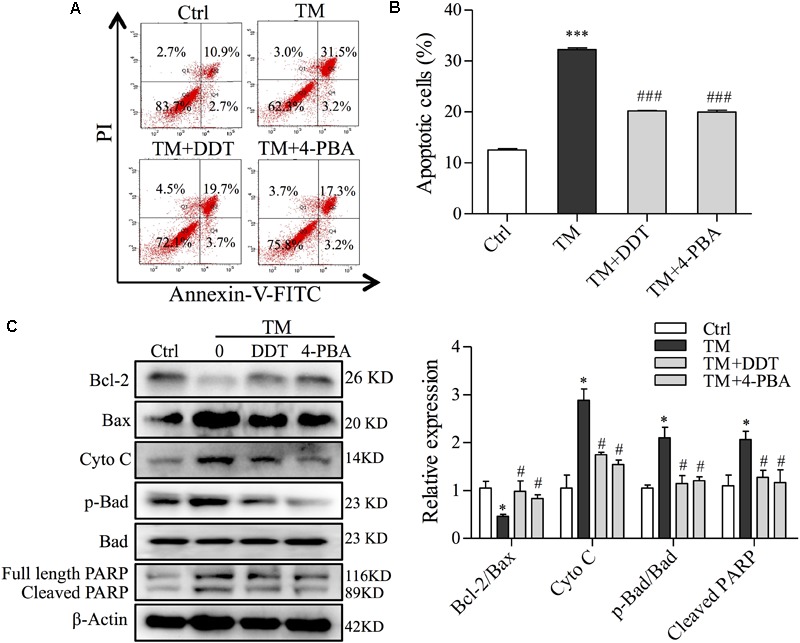
DiDang Tang inhibits ER stress-induced mitochondrial apoptosis in NGF-induced PC12 cells subjected to TM incubation. **(A)** After pretreatment with DDT or 4-PBA, followed by TM incubation, PC12 cells were stained with Annexin V-FITC/PI. We then detected the percentage of apoptotic cells by FCM. **(B)** Bar graph represents the number of apoptotic cells from **(A)** after the pretreatment with DDT or 4-PBA, followed by TM incubation. **(C)** Protein levels of Bcl-2, Bax, Cyto C, p-Bad, Bad and PARP were determined by Western blot. After the quantification by Image J, the intensity of each protein was normalized to the intensity of β-Actin and is shown on the right. ^∗^*P* < 0.05 and ^∗∗∗^*P* < 0.001 vs. Ctrl group; ^#^*P* < 0.05 and ^###^*P* < 0.001 vs. TM group (*n* = 3).

To further validate the protection of DDT on ER stress-induced mitochondrial apoptosis, we investigated the effect of DDT on mitochondrial apoptosis and apoptosis-related proteins induced by TM using FCM and Western blot analysis. Compared to the control, TM incubation largely increased the percentage of apoptosis from 13 to 32.3%. DDT and 4-PBA remarkably mitigated TM-induced apoptosis from 32.3 to 20.4% and 19.6%, respectively (Figures 8A,B). Moreover, DDT pretreatment increased the ratio of Bcl-2/Bax and reduced the levels of Cyto C, p-Bad/Bad, and cleaved PARP induced by TM exposure, which was similar to 4-PBA as shown in Figure 8C. Taken together, these data provide evidence that DDT inhibited PC12 cell death through decreasing TM-mediated mitochondrial dysfunction.

## Discussion

In ICH, chronic activation of ER stress has been considered as a main means of the pathogenesis of neuronal disorders (Duan et al., 2017). Therefore, substantial efforts have been investigated to identify anti-ER stress mediators with neuroprotective potential (Zhang et al., 2015). In this study, all of the results affirmed the strong neuroprotective potential of DDT against ER stress-mediated mitochondrial cell apoptosis by blocking the GRP78-IRE1/PERK pathways in neuronal cells and in rats, which could explain the molecular mechanism of DDT for the treatment of patients or animal models with ICH.

Endoplasmic reticulum stress-induced apoptosis is a key pathologic event of neuronal function during ICH (Eder et al., 2006). In response to OGD, neural cells adapted to the accumulation of unfolded or misfolded proteins in the ER lumen, which disturbed ER function to cause ER stress (Bravo et al., 2013). It is believed that ER chaperones, such as GRP78 and GRP94, regulate the responses to ER stress by inducing UPR proteins (Zhang et al., 2015). The activation of IRE1α and PERK involves an initial dissociation of BiP/GRP78 that drives sensor oligomerization and transphosphorylation, while subsequent binding to unfolded proteins leads to ER stress (Bravo et al., 2013). When misfolded ER proteins accumulate, PERK inhibits mRNA translation of new protein synthesis through the phosphorylation of eIF2α (Zhang et al., 2015). ATF6, an ER-membrane protein, is held inactive under normal conditions by the binding of GRP78 (Roussel et al., 2013). During stress, misfolded proteins sequester GRP78, which frees ATF6 to traffic to the Golgi apparatus, where it is cleaved by the Site-1 and then the Site-2 Proteases (Gan et al., 2017). In this study, significant protein build-ups of GRP78 and of three UPR-regulatory proteins were detected in OGD- and TM-treated neuronal cells and ICH rats. Pretreatment/treatment with DDT promoted the affinity of GRP78 toward its signaling partners, IRE1 and PERK, after OGD/TM incubation or ICH modeling. It is important to note that, DDT had no effect on the activation of ATF6 induced by OGD/TM incubation or ICH modeling, unlike 4-PBA, an inhibitor of ER stress, by blocking the GRP78-IRE1/ATF6/PERK pathways (Song et al., 2013). Therefore, it is plausible that DDT stabilized the interactions of GRP78 with IRE1 and PERK to restore ER homeostasis in ER stress-induced neuronal cells.

By localizing to both ER and mitochondria, Ca^2+^ and the Bcl-2 family may prevent apoptotic cross-talk between these two organelles (Cagalinec et al., 2016). The ER serves as the primary store in cells for Ca^2+^, a second messenger, participating in a wide variety of physiological functions (Shinde et al., 2016). It is reported that the accumulation of misfolded proteins in the ER lumen separates the chaperone from each sensor, along with Ca^2+^ release from the ER and caspase-dependent mitochondrial apoptotic damage (Tusi et al., 2011). Ca^2+^ sequestration by the ER is the main coordinator of intracellular Ca^2+^ homeostasis, and the loss of Ca^2+^ homeostasis during OGD is a hallmark of impending neuronal injury (Bodalia et al., 2013). Under OGD, ER stress evoked Ca^2+^ overload. Meantime, Ca^2+^-dependent folding and trafficking is also controlled by the chaperones responsible for proper protein folding in the ER (Krebs et al., 2011). Our data suggest that, upon ER stress stimuli, DDT is able to modulate Ca^2+^ homeostasis and regulates ER stress in a Ca^2+^-dependent manner in response to OGD stimulation. In the present study, we also proved that pretreatment with DDT abolished the decrease of MMP and the increase of Ca^2+^, as well as the increased levels of Cyto C and p-Bad after OGD incubation. Furthermore, we found a significant decrease of the ratio of Bcl-2/Bax in the OGD-treated cells, which was obviously increased in the DDT-pretreated groups. During apoptosis, reduced Bcl-2 and increased Bax can translocate from the cytoplasm to both mitochondria and the ER to interact with inositol-3-phosphate receptors (IP3R5) (Namba et al., 2013), which inhibits the release of Ca^2+^ from IP3R5 in the ER or increases the tolerance of mitochondria challenged with a high Ca^2+^ load (Huniadi et al., 2013; Pan et al., 2015). Based on the cross-talk between ER and mitochondria, our findings indicate that DDT could have protective effects on ER stress induced injury through inhibiting ER-dependent mitochondrial apoptosis.

However, current studies for DDT are limited to the holistic mixture. Although it is well-known that DDT is a classical Traditional Chinese Medicine and widely used in clinics for the treatment of ICH, the absorbed bioactive compositions derived from DDT that exert the protective functions remain unclear. Additional research for elucidating the molecular mechanisms underlying the anti-ER stress of bioactive components from DDT in a rat model of ICH will be our future direction.

In summary, for the first time, we have demonstrated that DDT pretreatment inhibited ER stress, Ca^2+^ overload, and consequent mitochondrial apoptosis in OGD- or TM-induced neuronal cells. Moreover, our results indicated that the neuroprotective effect of DDT against ER stress damage and apoptosis was mainly through the blockade of the GPR78-IRE1/PERK signaling pathways. The present investigation provides strong evidence that DDT could be a potentially promising therapeutic option for the treatment of ICH.

## Data Availability Statement

All data generated or analyzed during this study are included in this published article.

## Author Contributions

QH, XL, and JW conceived and designed the experiments. QH, TiaL, JL, and HZ performed the research. DZ and TiaL analyzed the data. XL and JW wrote the paper. PX, TinL, and JR drafted the manuscript. DZ and LS revised the manuscript. All authors gave the final approval and agreed to be accountable for all aspects of the work.

## Conflict of Interest Statement

The authors declare that the research was conducted in the absence of any commercial or financial relationships that could be construed as a potential conflict of interest.
