# Supplementary material for: DiDang Tang Inhibits Endoplasmic Reticulum Stress-Mediated Apoptosis Induced by Oxygen Glucose Deprivation and Intracerebral Hemorrhage Through Blockade of the GRP78-IRE1/PERK Pathways
Source: Front Pharmacol. 2018 Dec 4;9:1423. doi: 10.3389/fphar.2018.01423 (PMC6288198; doi:10.3389/fphar.2018.01423)
Supplement: Supplementary file 1 [file Table_1.docx]

**Table 1 Components of the DDT.**

| **English name** | **Chinese name** | **Latin name** | **Family** | **Voucher number** | **Weight (g)** | **Part used** |
| --- | --- | --- | --- | --- | --- | --- |
| Rhubarb | Da huang | Rheum palmatum L． | Polygonaceae | 170916 | 5 | Root and rhizome |
| Leech | Shui zhi | Whitmania pigra Whitman | Hirudinidae | 170824 | 3 | Whole animal |
| Peach seed | Tao ren | Semen persicae | Rosaceae | 171011 | 10 | Seed |
| Gadflies | Meng chong | Tabanus | Tabanidae | 171015 | 3 | Whole animal |
